# Ship Fire Detection Based on an Improved YOLO Algorithm with a Lightweight Convolutional Neural Network Model

**DOI:** 10.3390/s22197420

**Published:** 2022-09-29

**Authors:** Huafeng Wu, Yanglin Hu, Weijun Wang, Xiaojun Mei, Jiangfeng Xian

**Affiliations:** 1 Merchant Marine College, Shanghai Maritime University, Shanghai 201306, China; 2 College of Information Engineering, Shanghai Maritime University, Shanghai 201306, China; 3 Institute of Logistics Science and Engineering, Shanghai Maritime University, Shanghai 201306, China

**Keywords:** ship fire detection, YOLOv4-tiny, deep learning, lightweight model, SE attention mechanism, migration study

## Abstract

Ship fire is one of the greatest dangers to ship navigation safety. Nevertheless, typical detection methods have limited detection effectiveness and accuracy due to distance restrictions and ship motion. Although the issue can be addressed by image recognition algorithms based on deep learning, the computational complexity and efficiency for ship detection are tough. This paper proposes a lightweight target identification technique based on the modified YOLOv4-tiny algorithm for the precise and efficient detection of ship fires, taking into account the distinctive characteristics of ship fires and the marine environment. Initially, a multi-scale detection technique is applied to broaden the detection range and integrate deep semantic information, thereby enhancing the feature information of small targets and obscured objects and improving the detection precision. Then, the proposed algorithm employs the SE attention mechanism for inter-channel feature fusion to improve the capability of feature extraction and the precision of ship fire detection. Last but not least, picture transformation and migration learning are added to the small ship fire dataset to accelerate the convergence pace, improve the convergence effect, and reduce dataset dependence. The simulation experiments reveal that the proposed I-YOLOv4-tiny + SE model outperforms the benchmark algorithm in terms of ship fire detection accuracy and detection efficiency and that it satisfies the real-time ship fire warning criteria in demanding maritime environments.

## 1. Introduction

A total of 71% of the Earth’s surface is covered by the ocean, and the enormous water area produces natural canals. Since the 15th century, the fast expansion of shipping has made it possible for humans to move between continents, and the massive exchange of personnel and things has drastically affected the social and natural landscape. Until recently, 80–90 percent of global trade was conducted via maritime transit. As a form of maritime transportation, ships are prone to a number of accidents, including fire [1]. It is difficult to fight fires on ships because of the ship’s unique water environment and complex internal structure, including the ship’s cabin [1,2] and other key areas containing a large number of electronic equipment and combustible materials, numerous compartments, narrow passages, little room to maneuver, and limited firefighting equipment. According to available data, ship fires account for 11% of all ship accidents, placing them in fourth place; yet the amount of damage they cause ranks first among all maritime disasters. On 8 November 2010, the US luxury cruise ship “Glory Carnival” lost power due to a cabin fire, trapping 3299 passengers and 1167 crew members. On 28 December 2014, the Italian ferry “Norman Atlantic” caught fire while traveling in the Mediterranean near Greece, killing 10 people. In 2018, the Panamanian tanker “Sanchi” and the Hong Kong bulk carrier “CF CRYSTAL” collided. The accident caused a full-scale fire on board, with 3 deaths and 29 crew members missing. In China alone, 49 ship fire (explosion) incidents occurred between 2016 and 2020, resulting in 39 deaths and missing people, serious casualties, and huge property losses. The aforementioned incidents demonstrate that fires represent major navigational safety issues. In order to safeguard people’s lives and property, one of the critical responsibilities of ship safety navigation is to recognize and comprehend the ship fire scenario in a timely manner.

Currently, there are three basic methods for detecting fires: manual inspection, sensor detection, and image processing technologies. Traditional manual inspection and sensor detection have long response times, are easily fatigued, and are susceptible to interference from external factors such as spatial location, wind speed, temperature, and humidity, resulting in frequent false alarms and making early detection of fires difficult.

Image processing techniques offer the benefits of low cost and high efficiency and have steadily increased in terms of precision [3]. Consequently, a number of scientists have applied image processing techniques to recognize flames. For fire detection, traditional image processing techniques frequently employ manually selected features, such as color [4], texture [5], and geometric features [6], to segment fires, which are subsequently classified and matched using machine learning algorithms for fire detection. However, because of the complexity of the fire environment, typical image processing algorithms cannot match the requirements for model generalization capability and resilience in actual engineering through manually designed feature extraction.

Deep learning target detection can automatically extract image details and features, effectively overcoming the redundancy and interference caused by the manual extraction of image features [7]. For fire detection, fire recognition based on deep learning is an improvement over traditional image processing-based methods; hence, fire detection systems that rely on deep learning techniques rather than feature descriptions are gaining increasing attention [8,9].

Despite this, most deep learning fire video detection systems on the market require computers with powerful CPUs and GPUs to speed up computation as well as a few embedded platforms for recognizing images from the cloud. This is because of the emphasis on real-time recognition in target recognition and the relatively large amount of computation involved in deep learning. To begin with, they take up a great deal of space and money, require a great deal of wiring, and affect the layout of the hull. Good network coverage is required for the latter, even though it is small and inexpensive [10]. Consequently, neither can be utilized directly on ships.

This study provides a lightweight model for ship fire detection by employing the improved YOLOv4-tiny algorithm and taking ship-specific factors and engineering practicability into consideration. First, the dataset is constructed by collecting multiple fire images of a ship’s cabin, deck, and dwelling, and then the dataset is expanded by image transformation; second, the multi-scale detection strategy is applied to increase the feature extraction backbone network feature output layer to expand the detection range, and the K-means algorithm is used to cluster the labeled data samples to obtain the a priori frame parameters of different sizes. The SE attention mechanism is then used to determine whether or not a fire has occurred in a particular. Finally, migration learning is incorporated, and the test set is partitioned for testing on embedded devices to evaluate the detection performance of the proposed technique.

The remainder of the paper is structured as follows: Section 2 discusses the present development status of fire detection technologies and target detection techniques. Section 3 presents the improved method of a lightweight convolutional neural network for ship fire detection in detail. The details of the experimental environment settings, data preprocessing and model training are illustrated in Section 4. Section 5 discusses the comparison of experimental results, and finally, this paper concludes in Section 6.

## 2. Related Work

There are two main categories of fire detection techniques for visual recognition: traditional detection methods based on image features and detection methods based on deep learning. Previous research in visual recognition has relied more on feature extraction methods, such as flame-specific chromatograms, shapes, textures, flame motion, etc. A major problem of these traditional methods is the complex manual feature extraction task. Detection methods based on deep learning can automatically extract image details and features, effectively overcoming the redundancy and interference caused by manual image feature extraction. Therefore, the latest research extensively involves deep learning applications in detecting fires, and the results show higher accuracy and lower false alarm rates.

### 2.1. Traditional Fire Detection Methods Based on Image Features

Fire detection methods based on color features have been widely studied in the literature. Chen et al. [11] proposed a method using red channel thresholding for fire detection. Binti Zaidi et al. [12] performed fire detection based on RGB and YCbCr features of flames. Vipin et al. [13] used YBbCr color space to separate luminance from chromaticity and to determine whether pixels are fire regions are classified.

The texture feature extraction analysis of flames is also commonly used to detect and identify fires. Dimitropoulos et al. [14] constructed an SVM classifier based on motion, texture, flicker, and color features for fire detection. Ye et al. [15] proposed an implicit Markov tree and surface wave transform dynamic texture descriptor for fire detection by extracting flame texture features.

In addition to the literature considering color and texture features, some existing fire detection algorithms based on flame shape features and flame motion features have been applied. Yu et al. [16] proposed a fire detection algorithm using color and motion features. Li et al. [17] proposed a fire detection framework based on the color, dynamics, and flicker characteristics of the flame.

All of the aforementioned researchers have presented methods for extracting flame features, which have contributed to the advancement of visual fire detection and enhanced its precision. Due to the complexity of fire situations, the manual extraction of features is redundant and has a negative effect on detection accuracy.

### 2.2. Fire Detection Methods Based on Deep Learning

In recent years, SSD (single-shot multibox detector) [18], YOLO (You Only Look Once) [19], YOLO v2 [20], YOLO v3 [21], and YOLO v4 [22] for the first-stage networks and RCNN [23], Fast R-CNN [24], Faster R-CNN [25], and Mask R-CNN [26] for the second-stage networks have been successfully applied in many computer vision fields. Additionally, deep learning-based target detection methods have been widely used in many fire scenarios. For instance, Li P. et al. [27] used Faster R-CNN, YOLO, and SSD to detect indoor fires, and proposed an improved method based on YOLO to rise the accuracy. Wu H et al. [28] proposed an improved Faster R-CNN method for detecting and locating factory fire areas. Jiao et al. [29] proposed a forest fire detection model based on improved YOLOv3. Zhao, Lei et al. [30] proposed fire-YOLO model to detect small targets based on YOLO. Gagliardi et al. [31] proposed a faster region-based convolutional neural network (R-CNN) to detect suspicious fire regions (SRoF) and non-fire regions based on their spatial features. Abdusalomov et al. [32], combined with sensors, proposed a real-time high speed fire detection model based on YOLO. The above references, such as Ref. [27], can show experimentally that the image processing techniques based on deep learning theory have higher accuracy and lower missed detection rate compared to the traditional image processing techniques.

The above deep learning models improve the fire detection accuracy, but only for fires occurring on land, considering the unique ship environment, and most of the deep learning models require powerful hardware support and are not suitable for application on ships. Therefore, in this paper, we propose a multi-scale strategy and add an attention mechanism to the YOLOv4-tiny algorithm to further improve the detection accuracy, as it can both guarantee accuracy and adapt to the ship environment.

## 3. Methodology Ship Fire Detection Model Based on Improved YOLOv4-Tiny Network

**Figure 1:**
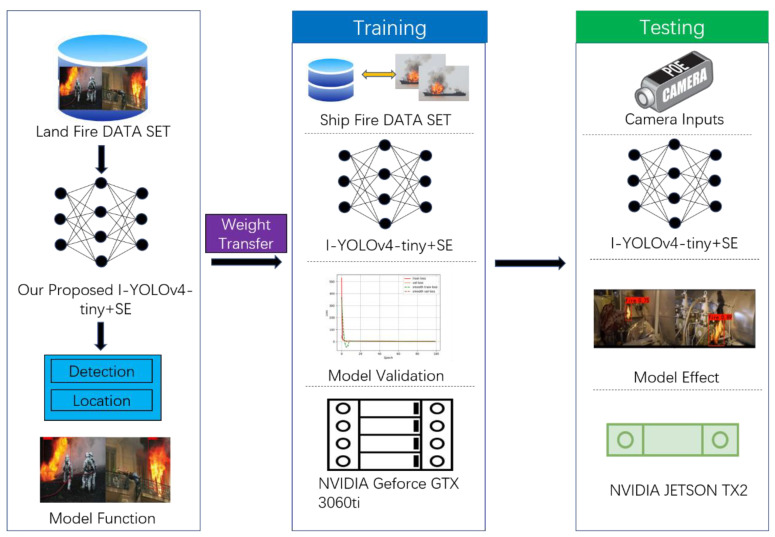
Flow chart of the proposed ship fire detection model based on improved YOLOv4-tiny network.

A ship fire detection model based on an enhanced YOLOv4-tiny network is proposed to identify ship fires and address the inadequacies of sensor detection and conventional image processing technology detection methods. Initially, the feature extraction backbone network feature output layer is added to the YOLOv4-tiny one-stage model to increase the detection range, and the I-YOLOv4-tiny model is shown. Adding the SE attention mechanism to the enhanced feature extraction network portion yields the I-YOLOv4-tiny + SENet model. Finally, the migration learning approach is implemented to lessen reliance on the ship fire dataset and expedite convergence in order to fulfill the objective of accurate ship fire detection. Figure 1 depicts the flowchart of the proposed ship fire detection model based on the upgraded YOLOv4-tiny network.

### 3.1. Introduction to YoLov4-Tiny Algorithm and Network Structure

**Figure 2:**
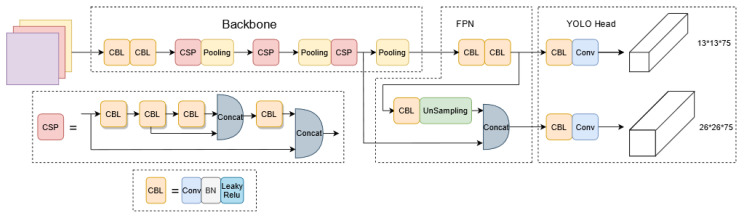
YOLO v4-tiny network structure.

YOLOv4-tiny is a single-stage target detection algorithm like YOLOv2, YOLO v3, and YOLO v4, but it is different in that it is more lightweight. At the beginning of the detection process, the images to be examined are divided into grids of different sizes. Each grid is accountable for a distinct region. If the target’s center falls within the grid, the grid is responsible for detecting the target. A backbone feature extraction network (Backbone), a feature pyramid network (FPN), and an output layer constitute the majority of the network structure (YOLO Head). The network architecture is depicted in Figure 2. Through the structure of a deep neural network, it extracts data features from the samples to make the trained model more suitable for identifying targets with complex properties.

### 3.2. I-YOLOv4-Tiny Lightweight Network Architecture

The YOLOv4-tiny model can detect targets and has a significant improvement in detection speed. However, the YOLOv4-tiny backbone network inputs the upgraded feature extraction network with just two feature layers, hence limiting the detection scale range. A multi-scale detection technique is proposed to handle the challenges of small flame and early flame targets of ships with weak characteristics and small size, which result in easily missed detection and false detection. This technique effectively increases the detection size range by extending the feature output layer of the feature extraction backbone network and combining the picture data from the deep and shallow layers.

The output layer of the YOLOv4-tiny model consists of two scale-size feature maps that have been downsampled by 16 (26 × 26) and 32 (13 × 13) times, respectively. After 16 times and 32 times downsampling, the spatial information contained in the feature layer loses a great deal of edge detail information, which can easily lead to the missed detection and false detection of small targets, whereas the second CSP structure of the backbone network contains a great deal of target detail features.

Consequently, the detection branch is added to the second CSP structure of the original YOLOv4-tiny backbone network, and feature extraction is performed on the feature map after 8 times (52 × 52) downsampling processing, which not only obtains more comprehensive target information, but also provides richer shallow feature information, thereby reducing the probability of missed and false detection caused by a large number of targets, small size, and partial occlusion. The 3 feature layers of 8 times, 16 times, and 32 times output from the backbone network are then output to the enhanced feature extraction network, where the feature information of 16 times downsampling is convolved and upsampled with the shallow features of 8 times downsampling to form a new scale for detection to obtain the improved I-Yolov4-tiny model.

**Figure 3:**
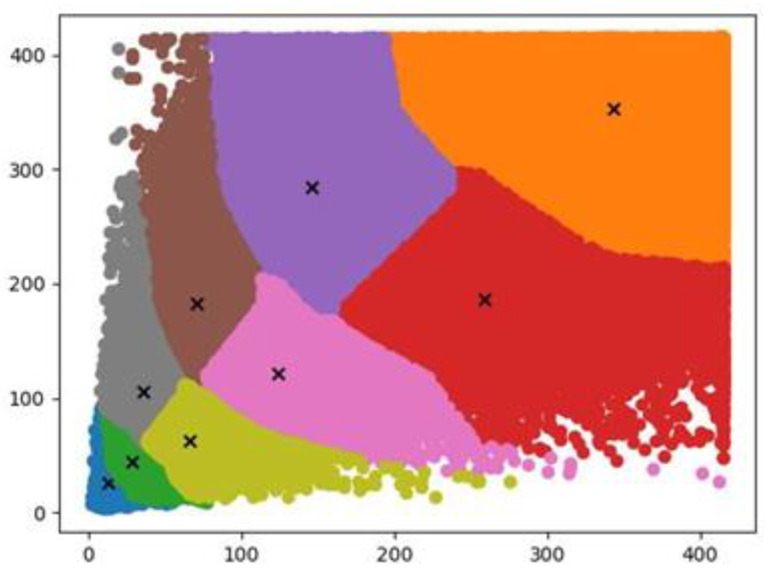
New anchor frame obtained by K-means algorithm clustering.

The I-Yolov4-tiny model adds one detection scale and increases the number of anchor frames from six to nine. Figure 3 depicts the outcomes of using the K-means algorithm to cluster and optimize the width and height of the training set targets to recover nine anchor frames.

**Figure 4:**
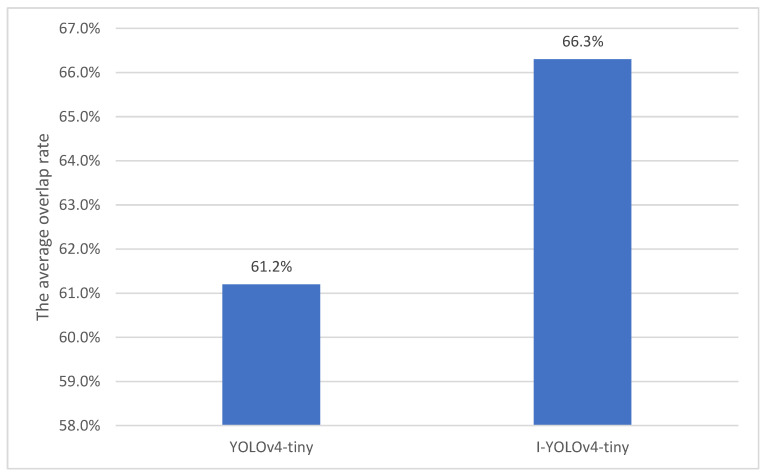
Comparison of the detection range between the improved model and the original model.

According to Figure 4, compared to the average overlap rate of six prediction frames in the original Yolov4-tiny model, the average overlap rate of nine prediction frames obtained by re-clustering with adding a detection scale is increased by 5.1%, bringing the model prediction closer to the original size of the target and enhancing the model’s detection range.

### 3.3. Building the I-YOLOv4-Tiny + SE Network—Introducing the Attention Mechanism

**Figure 5:**
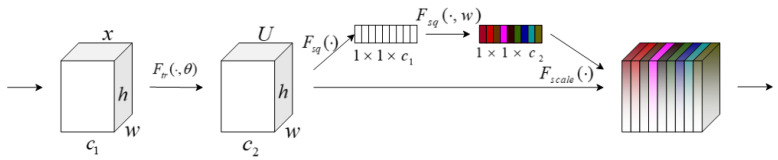
SE block structure.

Originally used for machine translation, the attention mechanism is now utilized in computer vision. By assigning different weights to its spatial and channel dimensions, a deep convolutional neural network (CNN) can be trained to focus on significant qualities and ignore irrelevant data. The SE (squeeze and excitation module) attention mechanism [33] is proposed to be employed in ship fire detection, as it is extensively used in target detection, but there does not appear to be any pertinent study for doing so. Figure 5 depicts the structure of the SE attention mechanism.

The SE attention mechanism is divided into the squeeze operation and excitation operation. First $F_{tr}$ is the conversion operation, which is a standard convolution operation in the text and plays the role of adjusting the number of channels. The input and output equations are expressed as follows:

(1) $$F_{tr}:X\to U,X\in {\mathbb{R}}^{W^{\prime }\times H^{\prime }\times C^{\prime }},U\in {\mathbb{R}}^{W\times H\times C}$$

Taking convolution as an example, the convolution kernel is $V=\left[v_{1},v_{2},\cdots ,v_{C}\right]$, $v_{C}$ denotes the cth convolution kernel. Then the output is $U=\left[u_{1},u_{2},\cdots ,u_{C}\right]$, and the formula is expressed as

(2) $$u_{c}=v_{c}*X=\sum_{s=1}^{c_{1}}v_{c}^{s}*X^{s}$$

where ∗ represents a convolution operation, and $v_{c}^{s}$ represents a two-dimensional convolution kernel with channels.

The second step is the squeeze operation; since the convolution only operates in a local space, which is difficult to obtain enough information to extract the relationship between channels. The phenomenon is more serious for the previous layers in the network due to its small perceptual field. For this reason, a squeeze operation is manipulated in the SE module, which encodes the entire spatial feature on a channel as a global feature, using global average pooling (GAP) to achieve

(3) $$z_{c}=F_{sq}\left(u_{c}\right)=\frac{1}{H\times W}\sum_{i=1}^{H}\sum_{j=1}^{W}u_{c}(i,j),z\in R^{C}$$

The excitation process is followed by the squeeze operation, which provides a global description of the characteristics. A third operation is then required to capture the interaction between channels. This operation must meet two criteria: first, it must be flexible and capable of learning the nonlinear relationships between the separate channels; second, the learned relationships cannot be mutually exclusive, as multichannel features are permitted in place of the single-channel form. Consequently, the sigmoid function is employed as the activation function here.

(4) s=Fex(z,W)=σ(g(z,W))=σ(W2ReLU(W1z))

The learned activation values (sigmoid activation, values 0 to 1) of each channel are then multiplied by the original features on U, making the model more discriminative of the characteristics of each channel. The weight coefficients for each channel are then learned as follows.

(5) $$\tilde{x}c=\;\mathrm{Fscale}\;\left(u_{c},s_{c}\right)=s_{c}\cdot u_{c}$$

“*c*” indicates the channel, “*s_c_*” indicates the channel weight vector, and “*u_c_*” indicates the channel feature map. Equation (5) is expressed as follows: the generated feature vector s is multiplied with the feature map u on the corresponding channel c, so that the feature map u is reassigned with weights on the channel, and the new feature map $\tilde{x}$ is obtained.

The SE attention mechanism module is added to the three feature input layers and two upsampling layers of the I-Yolov4-tiny model’s enhanced feature extraction network to perform feature fusion on the channels and improve the accuracy of ship flame identification.

The module for the SE attention mechanism is added to the I-YOLOv4-tiny model. By assigning weights to feature images on channels, computational resources are made more likely to focus on target regions, and channel-specific attention is filtered out to enhance the information of interest while suppressing irrelevant information, yielding good results, despite a slight increase in computational effort.

**Figure 6:**
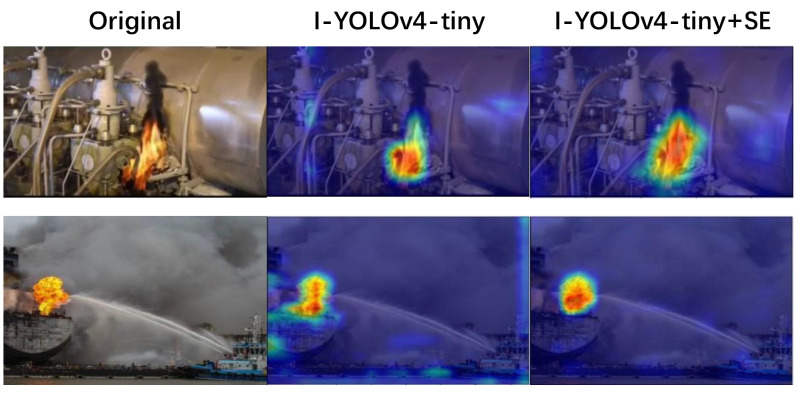
Grad-CAM flame heat map visualization results.

Using the Grad-CAM method [34] to compare the visualization results of the I-YOLOv4-tiny model with those of the I-YOLOv4-tiny + SE model, the experimental results presented in this research are depicted in Figure 6. It can be seen from the experimental results of the I-YOLOv4-tiny + SE model that the Grad-CAM mask covers the area of the target object well, and the effective prediction area has a larger range and more accurate results than the I-YOLOv4-tiny model. Moreover, it can be demonstrated that the SE attention mechanism for ship fire detection applications can learn and collect information from features in the target area.

**Figure 7:**
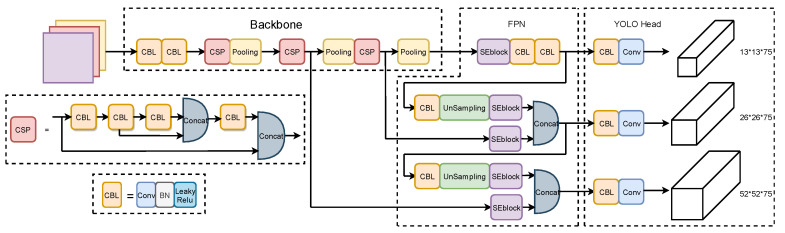
Overall structure of the improved lightweight network model.

The I-YOLOv4-tiny + SE model proposed in this paper uses YOLOv4-tiny as its foundational framework, proposes a multi-scale detection strategy, adds small-scale detection layers, and improves the detection of small flame targets on ships; additionally, the SE attention mechanism is added to the enhanced feature extraction network to improve the model’s ability to extract features and suppress invalid information, which further improves the detection accuracy of flame targets. Figure 7 depicts the general framework of the I-YOLOv4-tiny + SE model.

### 3.4. Introduction of Transfer Learning Methods

The convolutional neural network method for detecting ship fires has the following drawbacks: (1) The convolutional neural network model must be trained using a large number of training samples; otherwise, the model’s training will not have sufficiently improved the convolutional neural network method’s recognition performance. (2) It takes a long time to train a deep convolutional neural network model from scratch, and the more intricate its structure and the deeper its layers, the longer the training period will be required. (3) High-performance hardware platforms are expensive and require a lot of computational and storage power. The migrating learning method for ship fire detection is suggested as a solution to the issues with the current approaches.

**Figure 8:**
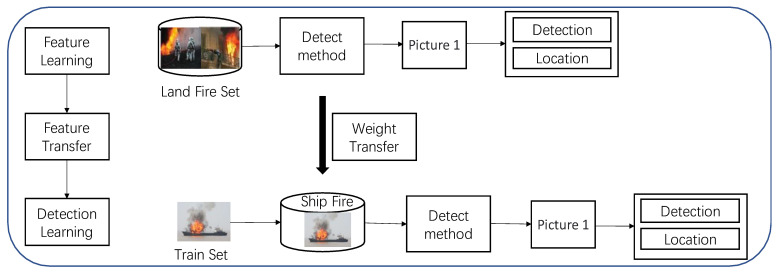
Migration learning process for ship fire detection.

In this study, two land fire datasets [5], fire set1 and fire set2 [35], are utilized as the base data; low-resolution photos are eliminated, and a new dataset is then created. The new dataset’s land fire images have a lot of flames underlying the information features because the underlying texture images are so common; as a result, the conditions for feature migration are present. The above land fire recognition task is thought to be well correlated with the ship fire recognition task. Thus, the pre-trained deep learning model based on the dataset of land fires can be migrated and used to the recognition of ship fires [36,37]. Figure 8 demonstrates the process of fire migration learning.

## 4. Experimental Environment Settings, Data Preprocessing, and Model Training

### 4.1. Experimental Environment and Evaluation Index

#### 4.1.1. Experimental Environment

The model provided in this study was tested in the laboratory, and the quantitative and qualitative outcomes were assessed. Pytorch, a deep learning framework, was used to train the model with an i7- 11700F CPU, Nvidia Geforce GTX 3060ti GPU, and 32 g of memory. To test the applicability of the proposed model for ship fire detection, the model was installed on a device with limited processing power and memory, the Nvidia Jetson TX2.

#### 4.1.2. Evaluation Index

This study draws P-R curves, presents mAP@.5 evaluation metrics, and FPS (detection speed) to evaluate the suggested model to validate the performance of the proposed I-YOLOv4-tiny + SE network model in ship fire detection.

(1) P-R curve, mAP@.5

Recall is the horizontal coordinate and accuracy is the vertical coordinate when plotting PR curves. If the PR curve of one model completely encircles the PR curve of another model, the former model is deemed to have superior performance. If this cannot be ascertained directly, the area under each model’s curve can be compared.

mAP@.5 is the computed target detection accuracy at IoU = 0.5. The average value of precision is an essential metric for evaluating models. AP is computed using precision and recall, and mAP is defined as the varied P and R that can be achieved when different confidence levels are selected, which are derived from Equations (6) and (7):

The accuracy (P) of the model is calculated by:

(6) $$P=\frac{\mathrm{TP}}{\mathrm{TP}+\mathrm{FP}}$$

The recall (recall, R) of the model is calculated as:

(7) $$R=\frac{\mathrm{TP}}{\mathrm{TP}+\mathrm{FN}}$$

As a result, the mean accuracy precision (mAP) is calculated as:

(8) $$\mathrm{mAP}=\int_{0}^{1}P(R)\mathrm{dR}$$

(2) Detection speed

The detection speed is a crucial need for real engineering applications, and we utilize the frame rate (FPS) to demonstrate the detection speed, which is a crucial metric for model evaluation. In general, if the FPS is below 30, the requirements are met, and the video detection function is smooth when the FPS is below 60.

### 4.2. Data Collection and Preprocessing

**Figure 9:**
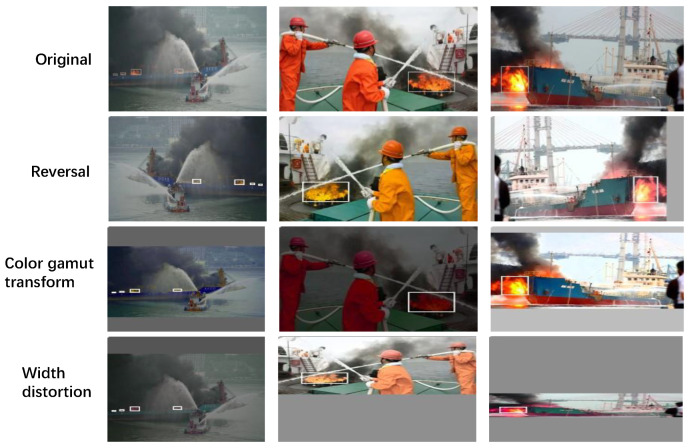
Example of ship fire dataset and image processing effect.

According to our understanding, there is no unified public dataset for ship fires; therefore, we develop a dataset for ship fire detection in this study. A web crawler was used to collect and delete photos with poor clarity. Before training the YOLO model, the collected photos were normalized and scaled down to 416 × 416 dimensions. After normalization, data are manually labeled using annotation. The labels’ file format is text. To improve the generalizability of the model and fully utilize and expand the dataset, the photos are inverted, aspect warped, and color gamut modified to enlarge the homemade dataset to 2160 images. Figure 9 displays the fire examples and impacts of image processing from the homemade ship fire dataset. It is randomly separated into the training set, validation set, and test set according to the 6:2:2 requirements of the experiment.

### 4.3. Model Training and Comparison

Following the concept of this paper, four algorithms, including YOLOv3-tiny [38], SSD [18], YOLOv4-tiny, and I-YOLOv4-tiny, were chosen as the comparison methods, and I-YOLOv4-tiny + SE were trained independently of the validation and test sets using the same training set. The YOLOv3-tiny, SSD and YOLOv4-tiny algorithms are advanced in the use of convolutional neural networks, thus they are widely employed in the field of fire detection. Numerous researchers in different specialized disciplines have successfully utilized multiscale detection fusion to target detection algorithms and obtained positive results. To accomplish this, we develop the I-YOLOv4-tiny network, incorporate an attention mechanism to improve detection accuracy, and then construct the I-YOLOv4-tiny + SE network. Finally, the research concludes by comparing and evaluating the performance of the I-YOLOv4-tiny + SE model proposed for ship fire applications.

**Figure 10:**
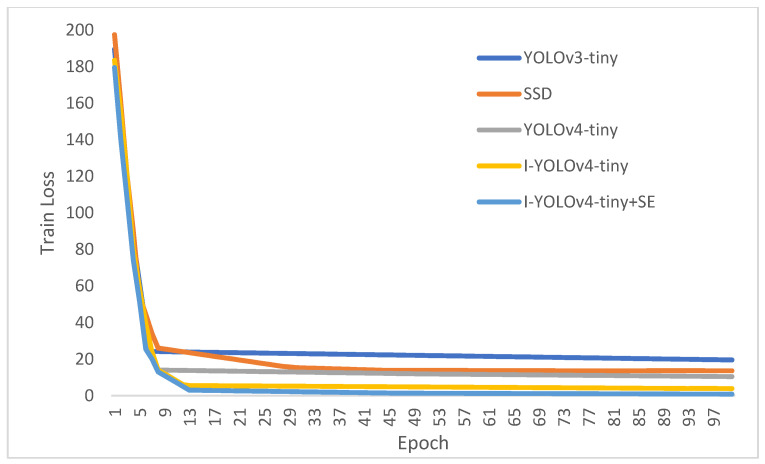
Comparison of ship fire training loss function curves for the five models.

We set the training time for this study to 100 and employed the Adam optimizer with 100 training rounds. For the first 50 rounds, the learning rate (learning rate) was 0.001, and for the final 50 rounds, it was 0.0001. We employed the migration learning technique to accelerate the convergence rate, which aids the model’s convergence loss. To apply the YOLO network structure, all input images must measure 416 by 416 pixels. The loss is the difference between predicted and actual values. As the gap gradually diminishes and converges, this indicates that the model is approaching the dataset’s maximum performance threshold. Figure 10 compares the training loss function curves for each of the five models.

As depicted in Figure 10, the loss values of all five models converge rapidly at the start of training, demonstrating that it is possible to apply migration learning approaches to accelerate convergence and reduce dataset dependence. After a predetermined number of iterations, the variability of the loss curve reduces progressively. Figure 10 illustrates that YOLOv3-tiny has worse convergence loss values. I-YOLOv4-tiny and I-YOLOv4-tiny + SE have comparable convergence values for their loss functions, and both outperform YOLOv4-tiny and SSD.

## 5. Results and Discussion

### 5.1. Evaluation Indicators

**Figure 11:**
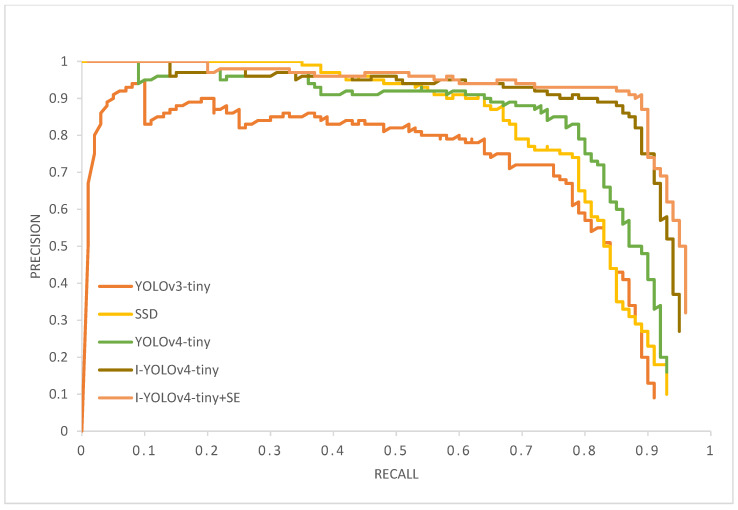
PR curves for the five fire detection models on the test set.

After training the five models, the models were assessed using a test dataset distinct from the training and validation sets. During testing, we divided the positive and negative samples by setting the IoU to 0.5 and plotting the PR curves for the performance of several models. As seen by the PR curves in Figure 11, the upgraded YOLOv4-tiny model outperforms its predecessors YOLOv3-tiny, SSD, and YOLOv4-tiny.

### 5.2. Evaluation Indicators

In Table 1, we summarize the performance of five deep learning network models on the ship fire test dataset and NVIDIA JETSON TX2 test experiments. Compared to the YOLOv3-tiny model, SSD, the YOLOv4-tiny model, and the I-YOLOv4-tiny model, the I-YOLOv4-tiny+ SE of mAP@.5 for the deep network learning model, increased by 19.5%, 10.9%, 8.5%, and 2.1%, respectively. Precision increased by 16.3 percent, 10.2 percent, 7.7 percent, and 1.9 percent, while recall increased by 22.1%, 18.1%, 9.2%, and 3.9%, respectively. I-YOLOv4-tiny + SE showed considerable gains in precision and recall over YOLOv3-tiny, SSD, and YOLOv4-tiny, demonstrating the efficacy of our approach. The multiscale fusion technique and the insertion of the attention mechanism reduce the processing speed of detection, but it is sufficient to meet the real-time detection criteria, as determined by an examination of the experimental results on detection speed. In conclusion, our suggested I-YOLOv4-tiny + SE deep network learning model beats SSD and the YOLOv4-tiny model in terms of mAP@.5, accuracy, recall, and other metrics across the board, and its detection speed metrics surpass those of another lightweight target detection YOLOv3-tiny network model.

We believe that the I-YOLOv4-tiny + SE model is more resilient in performance than the YOLOv3-tiny, SSD, YOLOv4-tiny, and I-YOLOv4-tiny network models, as determined by a complete comparison of the mAP@.5, precision, recall, and FPS evaluation metrics in the experimental data. The I-YOLOv4-tiny + SE model suggested in this study offers greater precision and adaptability, which is useful for embedded device deployment and practical applications.

**Figure 12:**
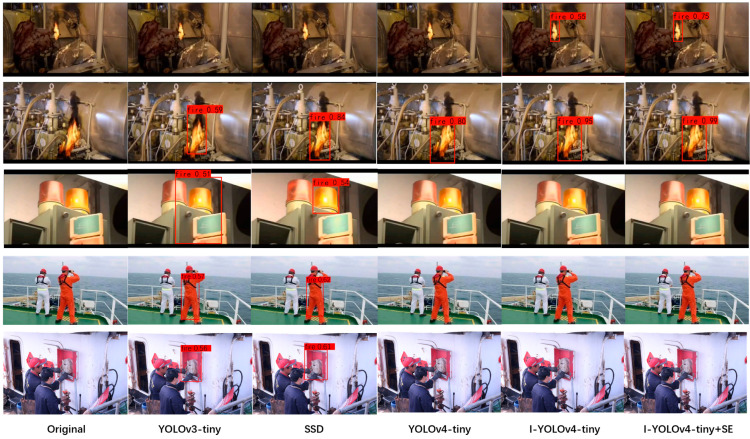
Detection results for small flame targets, large flame targets and fire-like targets.

As depicted in Figure 12, numerous fire scenarios, including small flame targets, enormous flames, and flame-like targets, were chosen for visual testing to comprehensively evaluate the performance of the I-YOLOv4-tiny + SE model.

As shown in Figure 12, the YOLOv3-tiny and SSD model was unable to detect the small flame target, and there were false detections for the fire-like target; the YOLOv4-tiny model was unable to detect the small flame target but could detect the large flame target, but there was no false detection for the fire-like target; and the I-YOLOv4-tiny model accurately detected the small flame target. The I-YOLOv4-tiny model identifies small flame targets and large flame targets without false detection; the I-YOLOv4-tiny + SE model increases the confidence level of small flame targets and large flame targets based on the I-YOLOv4-tiny model, resulting in a more precise detection effect.

Among all detection models, the YOLOv3-tiny model had the lowest confidence level, whereas the I-YOLOv4-tiny + SE model properly detected all flame targets and had the highest confidence level. This suggests that the multiscale fusion technique improves the model’s ability to recognize small objects, increases the recognition sensitivity of small targets, and decreases the probability of missed detections of targets with negligible features. In addition, it can be seen that by employing the attention mechanism to increase feature extraction, the detection accuracy of the model can be enhanced to some degree, resulting in improved performance of the model for ship fire detection. By conducting more performance tests on the enhanced model, we can confirm its performance advantages. In comparison to the YOLOv3-Tiny, SSD, YOLOv4-tiny, and I-YOLOv4-tiny versions, the I-YOLOv4-tiny + SE offers greater practical benefits. In the quantitative evaluation results and qualitative analysis, the I-YOLOv4-tiny + SE model proposed in this paper demonstrates strong anti-interference capability, high sensitivity to small targets, and low influence by external environmental interference, along with excellent robustness and generalizability.

## 6. Conclusions

In this research, we offer the I-YOLOv4-tiny + SE model for precise and fast ship fire detection in complex and dynamic marine situations. First, we constructed a high-standard and high-quality dataset on ship fires. Secondly, a multi-scale detection method was proposed to raise the feature output layer of the network’s backbone feature extraction layer to combine the picture information of deep and shallow layers and broaden the detection dimension range. The I-YOLOv4-tiny + SE network model introduces the attention mechanism in natural language processing, and by assigning different weights to the spatial dimension and channel dimension of the deep convolutional neural network (CNN), it enables the neural network to focus on focal features, thereby allowing more attention to be shifted to valid information. Under the same settings, we trained and evaluated the I-YOLOv4-tiny + SE, the YOLOv3-tiny, SSD, YOLOv4-tiny, and I-YOLOv4-tiny. The experimental results demonstrate that our proposed I-YOLOv4-tiny + SE model outperforms the deep learning models YOLOv3-Tiny, SSD, YOLOv4-tiny, and I-YOLOv4-tiny in terms of the mAP@.5 exponential by 19.5%, 10.9%, 8.5%, and 2.1%, respectively. In addition, the suggested model outperforms I-YOLOv4-tiny, YOLOv4-tiny, and YOLOv3-tiny, SSD in terms of precision and recall assessment indicators. On NVIDIA JETSON TX2, the detection speed was evaluated, and the FPS of the suggested model reached 51, which satisfies the detection requirements regularly. Next, we will examine utilizing separable convolution to minimize the number of parameters further, focus on loss to increase the accuracy, combine with knowledge of location information in different conditions [39,40,41,42], and try to use it in different usage scenarios, such as medical image detection [43], maritime search and rescue [44].

## Figures and Tables

**Table 1:** Test results of five detection models in the ship fire dataset.

Model	mAP@.5	Precision	Recall	FPS	Time (s)
YOLOv3-tiny [38]	0.711	0.765	0.654	45	0.022
SSD [18]	0.797	0.826	0.694	17	0.058
YOLOv4-tiny	0.821	0.851	0.783	68	0.014
I-YOLOv4-tiny	0.885	0.909	0.836	57	0.017
I-YOLOv4-tiny + SE	0.906	0.928	0.875	51	0.019

## Data Availability

The data presented in this study are available on request from the corresponding author. The data are not publicly available, as they involve the subsequent applications for patents, software copyright, and the publication of project deliverables.
